# Risk factors of developing contralateral breast cancer after first primary breast cancer treatment

**DOI:** 10.1002/cnr2.1927

**Published:** 2023-11-02

**Authors:** Maryam Avatefi, Fatemeh HadavandSiri, Seyed Saeed Hashemi Nazari, Mohammad Esmaeil Akbari

**Affiliations:** ^1^ Cancer Research Center Shahid Beheshti University of Medical Sciences Tehran Iran; ^2^ School of Public Health and Safety Shahid Beheshti University of Medical Sciences Tehran Iran

**Keywords:** breast cancer, contralateral, disease‐free interval, risk factors, second primary

## Abstract

**Background:**

Breast cancer (BC) is the most common cancer among women worldwide. Increased survival of primary BC (PBC) has increased contralateral breast cancer (CBC) and become a health problem.

**Aims:**

This study aimed to determine the effect of disease‐free interval (DFI), risk factors and PBC characteristics on the progression of CBC within primary BC survivors.

**Methods and Results:**

This retrospective study identified 5003 women diagnosed with breast cancer between 2000 and 2020 in the cancer research center. The study included 145 CBC and 4858 PBC survivors, with CBC diagnosed at least 6 months after the detection of primary BC. ER+, PR+, and HER2+ were reported in 72.13%, 66.67%, and 30% of CBC patients. Invasive ductal carcinoma (IDC) BC was reported in 69.57% of patients, and 81.90% and 83.64% of the patients were treated with adjuvant chemotherapy and external radiotherapy. The Kaplan–Meier method indicated that the median time interval between PBC and CBC was 3.92 years, and the 5‐year DFI was 97%. The Cox proportional hazard regression model indicated that although more than half of the participants had no family history of BC (69.57%), women 60 years and older were negatively associated with CBC.

**Conclusion:**

This study provides the first investigation of CBC and DFI risk factors among PBC survivors in Iran. Age was found to be negatively associated with CBC development particularly after the age of 60, indicating the necessity of tracking CBC survivors carefully in this age group.

## 1 INTRODUCTION

Breast cancer (BC) prognosis has improved in recent times. However, compared to other forms of cancer, primary breast cancer (PBC) is significantly associated with secondary cancer development.^1^ Contralateral breast cancer (CBC) is the term used to describe cancer in the opposite breast in a PBC survivor. CBC is typically considered a new and independent primary cancer rather than a metastasis from the initial primary.^2^

BC survivors have a higher incidence of CBC than the general population and it is the most common second cancer among BC patients.^3^, ^4^ Genetic susceptibility, long‐term effects of PBC treatment, and shared risk factors between the first and second BCs are likely to contribute to this increased risk.^5^ The number of PBC survivors is increasing, and the increased survival time has led to a significant increase in the chance of CBC.^6^

Second primary cancers can occur due to environmental risk, genetic, lifestyle, or treatment‐related factors.^7^ The most important risk factors for CBC include reproductive factors, lobular histology, mutation of BRCA1/BRCA2, and family history of BC.^8^ However, breast cancer is a heterogeneous disease, and environmental risk factors affect CBC development, leading to the necessity of studies within distinct environmental populations.

Although many studies have been conducted on the risk factors and epidemiology of breast cancer,^3^, ^9^ less research has been published on the related factors of CBC particularly within Iran. Therefore, this study aims to address the risk factors of developing CBC after treating the PBC in Iran. The study assesses the DFI of patients with PBC as well as the incidence, clinicopathologic features, and DFI of CBC, providing necessary insight into PBC monitoring.

## 2 MATERIALS AND METHODS

### 2.1 Study design and study population

In this retrospective cohort study, patient data were obtained from the cancer research center of Shahid Beheshti University of Medical Sciences in Tehran, Iran, from 2000 to 2020. The cancer research center registers breast cancer patients who undergo required investigations and routine clinical examinations. Following approval from the ethical committee of our institute, Shahid Beheshti University of Medical Sciences (No: IR.SBMU.CRC.REC.1400.048), we conducted a study to assess the risk factors of developing CBC including the interval between treatments of PBC after the identification of CBC or DFI. Based on prior reports, DFI is categorized into the first 5 years after the primary tumors detection (short DFI) and the following years (long DFI). Long DFI is associated with a better prognosis and favorable outcomes.^10^

### 2.2 Inclusion and exclusion

For this study, we followed patients treated for PBC to detect CBC and calculate DFI.

- The study focused on metachronous breast cancers, defined as tumors that manifested at least 6 months after the initial tumor diagnosis.^11^
- The patients who had synchronous or metachronous tumors at the time of admission were excluded from the study.
- Patients with bilateral synchronous BC (the presence of cancer in both breasts diagnosed within a short interval of time, usually within 3–6 months) were also excluded from the study.
- Individuals with a follow‐up period of less than 180 days were excluded from the study.

### 2.3 Data analysis

Descriptive analysis was conducted on the total sample of 5003 participants enrolled in the study. The chi‐square test was used for categorical variables, and the *t*‐test was used for continuous variables. Variables investigated include age, tumor size, ER and PR status, HER2 status, breast histology, chemotherapy, radiotherapy, lymph vascular invasion, family history, lymph node metastasis, hormone therapy, type of surgery, triple negative status, stage, and grade. All the variables with a *p*‐value less than .2 or most of the levels had a *p*‐value less than .2 in univariable analysis were included in the multivariable analysis.

The Kaplan–Meier curve was plotted to estimate the probabilities of 1‐, 5‐, 10‐, and 20‐year DFI. The proportional hazards assumption was tested using the Schoenfeld residual test followed by the Cox proportional hazard regression model was adopted to estimate the crude and adjusted hazard ratio (HR) and its confidence interval for the risk of CBC. The results of the multivariable analysis were expressed as HR and 95% CI. The significance level of 5% was considered for a statistically significant association.

Stata (version 14.0; Stata Corp., Texas, USA) software was used to for all statistical tests.

## 3 RESULTS

### 3.1 Study characteristics

A total of 5515 women diagnosed with PBC during the study's follow‐up time were reviewed. We excluded 375 bilateral synchronous breast cancer patients and 137 patients with incomplete profiles. In total, this allowed us to study 5003 PBC patients.

Of the selected patients, 145 (2.9%) progressed to CBC within the following years after treatment. The median age of these patients was 47 (range 17–78). All the other 4858 patients with a median age of 48 (range 17–90) were considered the PBC group. The median time interval between PBC and CBC was 3.92 years (confidence interval: 3.55–4.49). Of the patients who developed CBC, 36.92% were younger than 45 years old and more than half of the patients (57.65%) had a tumor size of 2–4 cm. Compared to patients with PBC, 61.54% of tumors in patients with CBC were 2–4 cm in size.

Invasive ductal carcinoma (IDC) is the most common type of breast cancer histology among CBC patients (69.57%). Of the CBC patients, 72.13% and 66.67% had ER+ and PR+ tumors, respectively, but 70% had negative HER2. For 74.63% of PBC, adjuvant chemotherapy was used. Nearly all (83.53%) subjects were treated with external radiotherapy, and 61.53% of patients had negative lymph vascular invasion. Of the patients with CBC, 69.57% did not have a family history of breast cancer (in first or second degrees). The prevalence of hormone therapy in patients with CBC was 89.62%. Of the patients who developed CBC, 45.26% had a mastectomy, and only 10.34% were triple negative (Table 1).

**TABLE 1:** Clinical characteristics of the unilateral and contralateral breast cancer in the study patients.

	*N* (%)			
Variables	Total (*N* = 5003)	BC (*N* = 4858)	CBC (*N* = 145)	*p*‐Value
Mean age at diagnosis (SD)	49.21 ± 11.77	49.28 ± 11.81	47.23 ± 9.96	.042
Median (range)	48 (17–90)	48 (17–90)	47 (17–78)	
Age				
<45	1681 (36.92)	1627 (36.86)	54 (38.85)	.03
45~	1991 (43.73)	1921 (43.52)	70 (50.36)	
>60	881 (19.35)	866 (19.62)	15 (10.79)	
Tumor size (cm)				
<2	1001 (27.00)	978 (27.14)	23 (22.12)	.52
2~	2137 (57.65)	2073 (57.54)	64 (61.54)	
>5	569 (15.35)	552 (15.32)	17 (16.35)	
ER status				
Positive	3023 (74.98)	2935 (75.06)	88 (72.13)	.46
Negative	1009 (25.02)	975 (24.94)	34 (27.87)	
PR status				
Positive	2771 (68.71)	2691 (68.77)	80 (66.67)	.62
Negative	1262 (31.29)	1222 (31.23)	40 (33.33)	
Her2 status				
Positive	893 (23.09)	860 (22.89)	33 (30.00)	.08
Negative	2974 (76.91)	2897 (77.11)	77 (70.00)	
Breast histology				
IDC	3426 (70.73)	3330 (70.76)	96 (69.57)	.17
DCIS	241 (4.98)	235 (4.99)	6 (4.35)	
ILC	286 (5.90)	272 (5.78)	14 (10.14)	
Others	891 (18.39)	869 (18.47)	22 (15.94)	
Chemotherapy				
No	355 (9.20)	342 (9.13)	13 (11.21)	.02
Adjuvant	2883 (74.63)	2788 (74.41)	95 (81.90)	
Neoadjuvant	625 (16.17)	617 (16.47)	8 (6.90)	
Radiotherapy (RT)				
No	105 (2.77)	100 (2.71)	5 (4.55)	.49
External RT	3170 (83.53)	3078 (83.53)	92 (83.64)	
IORT Boost	335 (8.83)	325 (8.82)	10 (9.09)	
IORT Radical	185 (4.87)	182 (4.94)	3 (2.73)	
Lymph vascular invasion (LVI)				
Positive	1417 (38.47)	1379 (38.47)	38 (38.78)	.95
Negative	2266 (61.53)	2206 (61.53)	60 (61.22)	
Family history				
No	2625 (72.62)	2545 (72.71)	80 (69.57)	.11
Second degree	529 (14.63)	505 (14.43)	24 (20.87)	
First degree	461 (12.75)	450 (12.86)	11 (9.57)	
Lymph node				
0	2135 (51.32)	2070 (51.22)	65 (54.62)	.28
1~	1077 (25.89)	1055 (26.11)	22 (18.49)	
4~	631 (15.17)	610 (15.10)	21 (17.65)	
>10	317 (7.62)	306 (7.57)	11 (9.24)	
Stage				
I	886 (21.10)	860 (21.09)	26 (21.31)	.84
II	1837 (43.75)	1781 (43.68)	56 (45.90)	
III, IV	1476 (35.15)	1463 (35.22)	40 (32.79)	
Grade				
I	426 (10.89)	413 (10.85)	13 (12.26)	.88
II	2132 (54.49)	2076 (54.53)	56 (52.83)	
III	1355 (34.62)	1318 (34.62)	37 (34.91)	
Hormone therapy				
Yes	3080 (88.28)	2985 (88.24)	95 (89.62)	.66
No	409 (11.72)	398 (11.76)	11 (10.38)	
Surgery type				
Breast‐conserving	3059 (69.41)	2984 (69.88)	75 (54.74)	<.001
Mastectomy	1348 (30.59)	1286 (30.12)	62 (45.26)	
Triple negative				
Yes	568 (11.35)	553 (11.38)	15 (10.34)	
No	4435 (88.65)	4305 (88.62)	130 (89.66)	.69

Abbreviations: BC, breast cancer; CBC, contralateral breast cancer; SD, standard deviation.

### 3.2 The DFI between PBC and CBC

**FIGURE 1:**
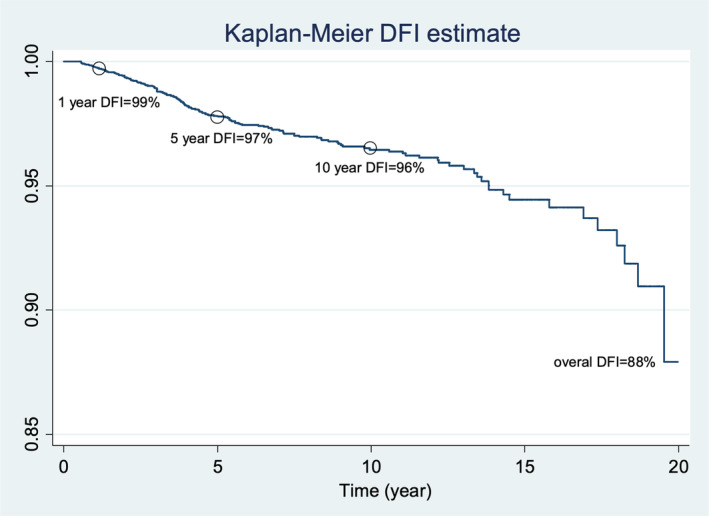
Kaplan–Meier curve of 1‐, 5‐, 10‐, and 20‐year disease‐free interval for contralateral breast cancer after primary breast cancer treatment.

**FIGURE 2:**
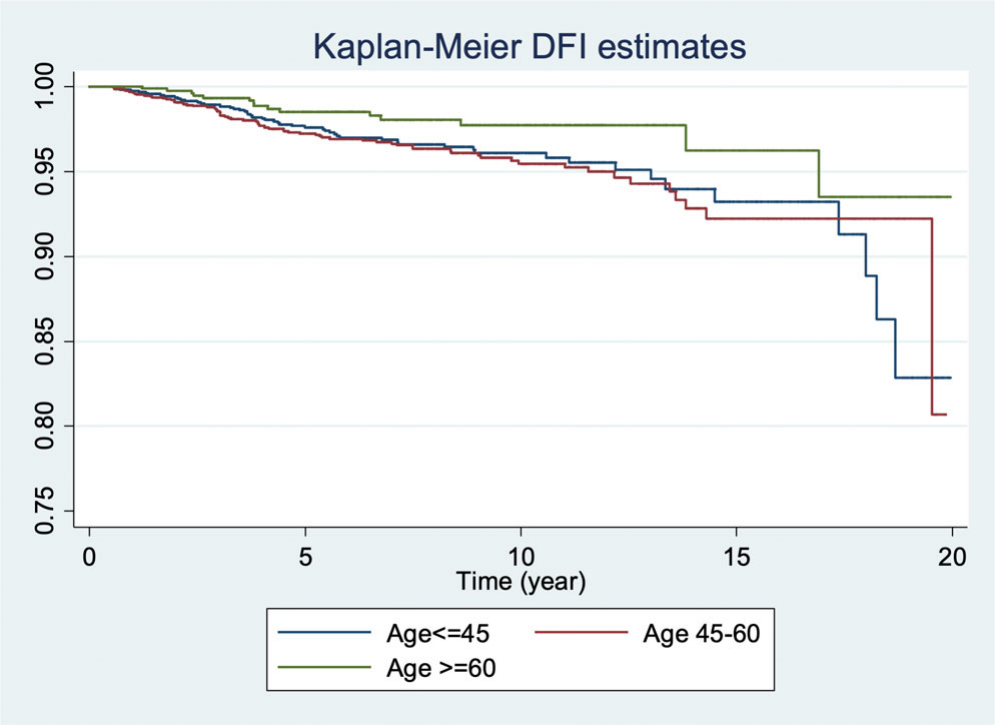
The overall disease‐free interval estimates among contralateral breast cancer patients by age.

For PBC, the probability of having a 1‐year DFI was 99%, while the probability for 5‐year, 10‐year, and 20‐year DFI was 97%, 96%, and 88%, respectively (Figure 1). The probability of DFI was higher in patients older than 60 years compared to other age groups (those under 45 years and those between 45 and 60 years; Figure 2).

Table 2 contains the results of univariable and multivariable Cox proportional hazards regression models. Most variables were protective. The age group of 60 years and higher (HR = 0.53; 95% CI: 0.30–0.94) and the IDC type of pathology (HR = 0.61; 95% CI: 0.38–0.97) were negatively associated with CBC progression. A history of breast cancer in the second degree was associated with a 59% increased likelihood of developing CBC (HR = 1.59; 95% CI: 1.01–2.51). The family history of breast cancer in the first degree was not associated with CBC. In multivariable analysis, after adjusting for covariates, the age group of 60 years and older (HR = 0.38; 95% CI: 0.16–0.87) was negatively associated with developing CBC. Additionally, 4–10 lymph node metastasis (HR = 0.91; 95% CI: 0.49–1.69) and >10 lymph node metastasis (HR = 0.69; 95% CI: 0.28–1.67) were negatively associated with developing CBC, but the results were not statistically significant.

**TABLE 2:** Hazard ratios for (multivariable cox regression analysis) CBC risk factors among patients.

Variables	Hazard ratios (95% confidence intervals)	
	Univariable model	Multivariable model
	HR (95% CI)	HR (95% CI)
Age		
<45	Ref.	Ref.
45~	1.05 (0.74–1.51)	0.93 (0.59–1.48)
>60	0.53* (0.30–0.94)	0.38 (0.16–0.87)
Tumor size (cm)		
<2	Ref.	‐
2~	1.18 (0.73–1.91)	‐
>5	1.04 (0.55–1.95)	‐
ER status		
Negative	Ref.	‐
Positive	0.97 (0.65–1.45)	‐
PR status		
Negative	Ref.	‐
Positive	0.93 (0.64–1.36)	‐
Her2 status		
Negative	Ref.	Ref.
Positive	1.32* (0.87–1.99)	1.05 (0.63–1.76)
Breast histology		
Others	Ref.	Ref.
IDC	0.61* (0.38–0.97)	0.63 (0.35–1.12)
DCIS	0.73 (0.29–1.82)	0.35 (0.04–2.92)
ILC	1.08 (0.55–2.12)	1.77 (0.78–4.01)
Chemotherapy		
No	Ref.	Ref.
Adjuvant	0.57* (0.31–1.02)	0.59 (0.28–1.26)
Neoadjuvant	0.40* (0.16–0.97)	0.39 (0.12–1.25)
Radiotherapy (RT)		
No	Ref.	‐
External RT	0.69 (0.28–1.70)	‐
IORT Boost	1.51 (0.50–4.48)	‐
IORT Radical	0.79 (0.18–3.35)	‐
Lymph vascular invasion (LVI)		
Negative	Ref.	‐
Positive	0.95 (0.63–1.43)	‐
Family history		
No	Ref.	Ref.
Second degree	1.59* (1.01–2.51)	1.67 (0.96–2.89)
First degree	0.84 (0.44–1.58)	1.00 (0.50–1.97)
Lymph node metastasis		
0	Ref.	Ref.
1~	0.65* (0.40–1.05)	0.64 (0.36–1.16)
4~	0.93 (0.56–1.52)	0.91 (0.49–1.69)
>10	0.94 (0.49–1.78)	0.69 (0.28–1.67)
Stage		
I	Ref.	‐
II	0.98 (0.61–1.56)	‐
III, IV	0.88 (0.53–1.44)	‐
Grade		
I	Ref.	‐
II	0.89 (0.48–1.63)	‐
III	0.95 (0.50–1.80)	‐
Hormone therapy		
No	Ref.	
Yes	1.01 (0.93–1.09)	‐
Surgery type		
Breast‐conserving	Ref.	
Mastectomy	1.28* (0.91–1.81)	1.46 (0.90–2.37)
Triple negative		
No	Ref.	
Yes	0.91 (0.53–1.55)	‐

Abbreviation: CBC, contralateral breast cancer.

* *p*‐Value less than .2 included in the multivariate analysis.

Table 3 presents the cumulative incidence results of CBC. After 4 years of follow‐up, the cumulative incidence of CBC was 1.4% for IDC, 1.5% for DCIS, and 3.5% for ILC.

**TABLE 3:** Cumulative incidence of CBC by histology of BC patients.

Time	Total	Fail	Failure function	Standard error	95% CI
*Other*					
0	0	0	0.000	‐	‐
2	618	5	0.006	0.002	0.002–0.015
4	375	9	0.024	0.006	0.014–0.040
6	242	4	0.036	0.009	0.022–0.059
8	153	3	0.052	0.012	0.032–0.084
10	100	1	0.062	0.015	0.037–0.101
12	60	0	0.062	0.015	0.037–0.101
14	19	0	0.062	0.015	0.037–0.101
16	13	0	0.062	0.015	0.037–0.101
18	6	0	0.062	0.015	0.037–0.101
20	1	0	‐	‐	‐
*IDC*					
0	0	0	0.000	‐	‐
2	3116	16	0.004	0.001	0.003–0.008
4	2685	29	0.014	0.002	0.011–0.019
6	2264	19	0.022	0.002	0.017–0.028
8	1722	8	0.026	0.003	0.020–0.033
10	1203	7	0.030	0.003	0.024–0.038
12	772	4	0.034	0.004	0.027–0.043
14	459	7	0.046	0.006	0.036–0.059
16	234	3	0.054	0.007	0.041–0.072
18	133	1	0.059	0.009	0.044–0.080
20	4	2	‐	‐	‐
*DCIS*					
0	0	0	0.000	‐	‐
2	183	2	0.008	0.006	0.002–0.034
4	144	1	0.015	0.009	0.004–0.048
6	126	1	0.022	0.011	0.008–0.061
8	92	0	0.022	0.011	0.008–0.061
10	54	0	0.022	0.011	0.008–0.061
12	28	0	0.022	0.011	0.008–0.061
14	14	1	0.071	0.048	0.018–0.257
16	10	0	0.071	0.048	0.018–0.257
18	3	0	0.071	0.048	0.018–0.257
20	1	1	‐	‐	‐
*ILC*					
0	0	0	0.000	‐	‐
2	254	4	0.014	0.007	0.005–0.038
4	232	5	0.035	0.011	0.018–0.066
6	201	3	0.048	0.013	0.027–0.083
8	171	1	0.053	0.014	0.031–0.089
10	117	1	0.059	0.015	0.035–0.099
12	60	0	0.059	0.015	0.035–0.099
14	26	0	0.059	0.015	0.035–0.099
16	7	0	0.059	0.015	0.035–0.099
18	4	0	0.059	0.015	0.035–0.099
20	1	0	‐	‐	‐

Abbreviations: BC, breast cancer; CBC, contralateral breast cancer.

## 4 DISCUSSION

Despite extensive research on breast cancer that has led to successful treatment and increased survival rates, the development of CBC after treatment of PBC remains a major issue that requires further research.

The present study found that the mean age of patients was 49 years, which is in contrast to another study that reported a mean age of 61.2 years at the first breast cancer diagnosis.^5^ The median time interval between PBC and CBC was 3.92 years, which differs from other studies that reported median time intervals of 6.2–6.7 years.^12^, ^13^ These differences in median age and time interval between PBC and CBC may be due to differences in the population's age composition. Previous research suggests that people with PBC can develop CBCs more than 5 years after diagnosis.^13^ The reported incidence of CBC was 3% after 5 years, which is comparable to other studies, which range in rate from 3% to 41.5%.^3^, ^12^ The possible reasons for differences in the frequency of incidence of CBC in the present and previous studies could be due to several factors, including differences in study design, patient characteristics, follow‐up duration, or differences in treatments.

Our study found that patients older than 60 had a lower chance of developing CBC. There is inconclusive reports regarding the effect of age on the progression of CBC, with some studies reporting a positive association between age and CBC and some reporting a negative association.^3^, ^12^, ^14^ We believe our results are an accurate representation of the young age pyramid in Iran, where diagnosed CBC was more common among younger people.

In the present study, a family history of BC in the second degree was associated with a 59% increased risk of CBC, while the family history of BC among first‐degree relatives was not associated with an increased risk of developing CBC. This finding is consistent with Yoon et al and Vichapat et al.^15^, ^16^ However, other studies have suggested that women with a strong family history of BC are at higher risk of developing CBC.^17^, ^18^

Consistent with the inconclusive evidence of HER2+ association with CBC, this study found no association between the two.^15^, ^19^, ^20^, ^21^

Also consistent with literature, we found that radiotherapy had no statistically significant effect on the incidence of CBC.^22^ However, another study found contradictory results.^23^ Of course, this is related to a kind of surgery such as mastectomy or saving the breast. Hence, it is not a scientific statement about irradiation effectiveness. In Yadav et al's study,^18^ adjuvant chemotherapy had no significant effect on the risk of second malignancy. In Alkner et al, chemotherapy given after BC made a difference in the progression of the disease and disease‐free interval (DFI). In this study, chemotherapy after BC was a negative prognostic factor.^13^ In the present study, adjuvant or neoadjuvant chemotherapy was not significantly protective or promotive for the CBC.

Although invasive lobular carcinoma is the second most prevalent type of breast cancer pathology,^24^ there are still conflicting results about its effect on CBC. According to a study by Tong et al,^25^ patients with invasive lobular carcinoma or a mixture of IDC and invasive lobular carcinoma had a higher likelihood of developing CBC compared to IDC alone.^24^ Glas et al revealed a higher risk of CBC in patients with lobular tumor morphology.^26^ In our study, the ILC was 10.14% versus 5.78% in CBC and PBC groups, respectively, and IDC was a protective factor for CBC.

Breast cancer, as a significant health concern in Iran, has an age‐standardized incidence rate (ASR) of about 34.53 per 100 000 in 2014.^27^ This rate is lower than the global age‐standardized incidence rate of breast cancer in females, estimated to be 48/100 000.^28^ According to the results of immunohistochemical studies, 10%–20% of breast cancer cases are triple‐negative. This subtype is characterized by significant proliferative activity and growth rate aggressive clinical course. Triple‐negative breast cancer (TNBC) is a specific subtype of breast cancer that does not express estrogen receptor (ER), progesterone receptor (PR), or human epidermal growth factor receptor 2 (HER‐2). TNBC has clinical features that include high invasiveness, metastatic potential, relapse potential, and poor prognosis.^29^, ^30^ In our study, 11.35% of patients were triple‐negative; of those, 2.64% developed CBC.

This is the first study of CBC in Iran that included data from a follow up period of 20 years. One limitation of this study is the lack of access to demographic variables such as reproductive, lifestyle, and death criteria in order to adjust for those potentially confounding risk factors. Furthermore, the lack of significance in several variables may be attributed to the limited sample size and the absence of measurement or recognition of potential confounding factors and study conditions. In future studies, we plan to employ a larger number of participants and obtain all demographic variables of the BC patient.

## 5 CONCLUSION

This study was the first to investigate CBC and DFI risk factors among BC survivors in Iran. This information will help clinicians identify patients at higher risk of CBC and develop appropriate surveillance and treatment plans. By identifying risk factors for CBC and DFI, patient outcomes will be improved by enabling more personalized treatment plans for high‐risk patients who may benefit from more aggressive treatment or closer surveillance.

## AUTHOR CONTRIBUTIONS

**Maryam Avatefi:** Reviewed the paper and edited the paper. **Fatemeh HadavandSiri:** Performed the analytic calculations and interpretation and wrote original draft preparation, review and editing. **Fatemeh HadavandSiri, Seyed Saeed Hashemi Nazari:** Developed the conceptual framework and verified the analytical methods. **Seyed Saeed Hashemi Nazari:** Review and editing. **Mohammad Esmaeil Akbari**: Supervised the project and edited the paper.

## CONFLICT OF INTEREST STATEMENT

The authors declare no conflict of interest.

## ETHICS STATEMENT

Ethical permission for the present study was obtained from the ethical committee of the cancer research center of Shahid Beheshti University of Medical Sciences (No: IR.SBMU.CRC.REC.1400.048). Verbal informed consent was obtained from patients.

## Data Availability

The data that supported the present study's findings are not publicly available. The data are only available from the corresponding author for approved proposals on reasonable request.

## References

[1] Youlden DR , Baade PD . The relative risk of second primary cancers in Queensland, Australia: a retrospective cohort study. BMC Cancer. 2011;11:83. 21342533 10.1186/1471-2407-11-83 PMC3052198

[2] Begg CB , Ostrovnaya I , Geyer FC , et al. Contralateral breast cancers: independent cancers or metastases? Int J Cancer. 2018;142(2):347‐356. 28921573 10.1002/ijc.31051 PMC5749409

[3] Ramin C , Withrow DR , Davis Lynn BC , Gierach GL , Berrington de González A . Risk of contralateral breast cancer according to first breast cancer characteristics among women in the USA, 1992‐2016. Breast Cancer Res. 2021;23(1):24. 33596988 10.1186/s13058-021-01400-3 PMC7890613

[4] Reiner AS , Sisti J , John EM , et al. Breast cancer family history and contralateral breast cancer risk in young women: an update from the women's environmental cancer and radiation epidemiology study. J Clin Oncol. 2018;36(15):1513‐1520. 29620998 10.1200/JCO.2017.77.3424 PMC5959199

[5] Feigelson HS , Bodelon C , Powers JD , et al. Body mass index and risk of second cancer among women with breast cancer. J Natl Cancer Inst. 2021;113(9):1156‐1160. 33823007 10.1093/jnci/djab053 PMC8757319

[6] Zheng X , Li X , Wang M , et al. Second primary malignancies among cancer patients. Ann Transl Med. 2020;8(10):638. 32566575 10.21037/atm-20-2059 PMC7290649

[7] Oeffinger KC , Baxi SS , Novetsky Friedman D , Moskowitz CS . Solid tumor second primary neoplasms: who is at risk, what can we do? Semin Oncol. 2013;40(6):676‐689. 24331190 10.1053/j.seminoncol.2013.09.012 PMC3921623

[8] Brooks JD , John EM , Mellemkjær L , et al. Body mass index, weight change, and risk of second primary breast cancer in the WECARE study: influence of estrogen receptor status of the first breast cancer. Cancer Med. 2016;5(11):3282‐3291. 27700016 10.1002/cam4.890 PMC5119984

[9] Ramin C , Mullooly M , Schonfeld SJ , et al. Risk factors for contralateral breast cancer in postmenopausal breast cancer survivors in the NIH‐AARP diet and health study. Cancer Causes Control. 2021;32(8):803‐813. 33877513 10.1007/s10552-021-01432-2 PMC8499045

[10] Van Laar C , Van Der Sangen M , Poortmans P , et al. Local recurrence following breast‐conserving treatment in women aged 40 years or younger: trends in risk and the impact on prognosis in a population‐based cohort of 1143 patients. Eur J Cancer. 2013;49(15):3093‐3101. 23800672 10.1016/j.ejca.2013.05.030

[11] Padmanabhan N , Subramanyan A , Radhakrishna S . Synchronous bilateral breast cancers. J Clin Diagn Res. 2015;9(9):XC05. 10.7860/JCDR/2015/14880.6511 PMC4606324 26500995

[12] Liederbach E , Piro R , Hughes K , Watkin R , Wang CH , Yao K . Clinicopathologic features and time interval analysis of contralateral breast cancers. Surgery. 2015;158(3):676‐685. 26067460 10.1016/j.surg.2015.03.059

[13] Alkner S , Bendahl P‐O , Fernö M , Manjer J , Rydén L . Prediction of outcome after diagnosis of metachronous contralateral breast cancer. BMC Cancer. 2011;11:114. 21450091 10.1186/1471-2407-11-114 PMC3080341

[14] Nichols HB , Berrington de González A , Lacey JV Jr , Rosenberg PS , Anderson WF . Declining incidence of contralateral breast cancer in the United States from 1975 to 2006. J Clin Oncol. 2011;29(12):1564‐1569. 21402610 10.1200/JCO.2010.32.7395 PMC3082975

[15] Yoon TI , Kwak BS , Yi OV , et al. Age‐related risk factors associated with primary contralateral breast cancer among younger women versus older women. Breast Cancer Res Treat. 2019;173(3):657‐665. 30377870 10.1007/s10549-018-5031-4

[16] Vichapat V , Gillett C , Fentiman IS , Tutt A , Holmberg L , Lüchtenborg M . Risk factors for metachronous contralateral breast cancer suggest two aetiological pathways. Eur J Cancer. 2011;47(13):1919‐1927. 21658939 10.1016/j.ejca.2011.05.004

[17] Lizarraga IM , Sugg SL , Weigel RJ , Scott‐Conner CE . Review of risk factors for the development of contralateral breast cancer. Am J Surg. 2013;206(5):704‐708. 24016706 10.1016/j.amjsurg.2013.08.002

[18] Yadav BS , Sharma SC , Patel FD , Ghoshal S , Kapoor RK . Second primary in the contralateral breast after treatment of breast cancer. Radiother Oncol. 2008;86(2):171‐176. 17961777 10.1016/j.radonc.2007.10.002

[19] Bessonova L , Taylor TH , Mehta RS , Zell JA , Anton‐Culver H . Risk of a second breast cancer associated with hormone‐receptor and HER2/neu status of the first breast cancer. Cancer Epidemiol Prev Biomark. 2011;20(2):389‐396. 10.1158/1055-9965.EPI-10-1016 PMC4110060 21217087

[20] Kheirelseid EA , Jumustafa H , Miller N , et al. Bilateral breast cancer: analysis of incidence, outcome, survival and disease characteristics. Breast Cancer Res Treat. 2011;126(1):131‐140. 20665107 10.1007/s10549-010-1057-y

[21] Mruthyunjayappa S , Zhang K , Zhang L , Eltoum IA , Siegal GP , Wei S . Synchronous and metachronous bilateral breast cancer: clinicopathologic characteristics and prognostic outcomes. Hum Pathol. 2019;92:1‐9. 31351153 10.1016/j.humpath.2019.07.008

[22] Drooger JC , Akdeniz D , Pignol J‐P , et al. Adjuvant radiotherapy for primary breast cancer in BRCA1 and BRCA2 mutation carriers and risk of contralateral breast cancer with special attention to patients irradiated at younger age. Breast Cancer Res Treat. 2015;154:171‐180. 26467044 10.1007/s10549-015-3597-7 PMC4621694

[23] Stovall M , Smith SA , Langholz BM , et al. Dose to the contralateral breast from radiotherapy and risk of second primary breast cancer in the WECARE study. Int J Radiat Oncol Biol Phys. 2008;72(4):1021‐1030. 18556141 10.1016/j.ijrobp.2008.02.040 PMC3782859

[24] Langlands F , White J , Kearins O , et al. Contralateral breast cancer: incidence according to ductal or lobular phenotype of the primary. Clin Radiol. 2016;71(2):159‐163. 26703116 10.1016/j.crad.2015.10.030

[25] Tong J , Tan D , Ma J , Hu Y , Li M . Nomogram to predict contralateral breast cancer risk in breast cancer survivors: a SEER‐based study. Medicine (Baltimore). 2021;100(46):e27595. 34797281 10.1097/MD.0000000000027595 PMC8601336

[26] de Glas NA , Engels CC , Bastiaannet E , et al. Contralateral breast cancer risk in relation to tumor morphology and age‐in which patients is preoperative MRI justified? Breast Cancer Res Treat. 2015;150(1):191‐198. 25677741 10.1007/s10549-015-3294-6 PMC4344552

[27] Roshandel G , Ghanbari‐Motlagh A , Partovipour E , et al. Cancer incidence in Iran in 2014: results of the Iranian National Population‐based Cancer Registry. Cancer Epidemiol. 2019;61:50‐58. 31132560 10.1016/j.canep.2019.05.009

[28] Wilkinson L , Gathani T . Understanding breast cancer as a global health concern. Br J Radiol. 2022;95(1130):20211033. 34905391 10.1259/bjr.20211033 PMC8822551

[29] Wang Q , Xu M , Sun Y , et al. Gene expression profiling for diagnosis of triple‐negative breast cancer: a multicenter, retrospective cohort study. Front Oncol. 2019;9:354. 31134153 10.3389/fonc.2019.00354 PMC6513966

[30] Kumar P , Aggarwal R . An overview of triple‐negative breast cancer. Arch Gynecol Obstet. 2016;293:247‐269. 26341644 10.1007/s00404-015-3859-y

